# Diagnostic performance and reported diagnostic thresholds of serum and synovial host-response biomarkers for periprosthetic joint infection in patients with inflammatory arthritis: protocol for a systematic review and diagnostic test accuracy meta-analysis

**DOI:** 10.3389/fmed.2026.1936572

**Published:** 2026-10-27

**Authors:** Jixin Chen, Qinxin Zhou, Yongming Zhang, Jianliang Chen, Xiaodong Zheng, Feng Ye

**Affiliations:** 1 Department of Orthopaedic Surgery, Shaoxing Shangyu District Hospital of Traditional Chinese Medicine, Shaoxing, China; 2 Department of Orthopaedic Surgery, Shaoxing Hospital of Traditional Chinese Medicine Affiliated to Zhejiang Chinese Medical University, Shaoxing, China

**Keywords:** diagnostic accuracy, host-response biomarkers, inflammatory arthritis, meta-analysis, periprosthetic joint infection, rheumatoid arthritis, synovial fluid, threshold

## Abstract

**Background:**

Periprosthetic joint infection (PJI) can sometimes be difficult to diagnose in patients with inflammatory arthritis because autoimmune disease activity and immunomodulatory treatment can alter systemic and synovial inflammatory responses. Diagnostic thresholds derived predominantly from patients with osteoarthritis may therefore produce false-positive or false-negative classifications in this population. Recent rheumatoid arthritis-specific cohorts and studies of synovial host-response biomarkers have expanded the evidence base beyond that available in earlier reviews.

**Methods:**

We will conduct a systematic review and diagnostic test accuracy meta-analysis of studies evaluating serum, plasma, whole-blood, or synovial host-response biomarkers for hip or knee PJI in adults with inflammatory arthritis. We will search MEDLINE, Embase, Web of Science, Scopus, CINAHL, the Cochrane Library, CNKI, Wanfang, VIP, and SinoMed from inception without language restrictions. Two reviewers will independently screen records, extract data, and assess risk of bias and applicability using QUADAS-3; an adapted QUADAS-C assessment will additionally be used for direct comparative accuracy studies. For each biomarker and clinically coherent threshold group, paired sensitivity and specificity will be synthesized using bivariate random-effects models or hierarchical summary receiver operating characteristic models. Threshold effects, reference-standard incorporation bias, joint type, infection chronicity, disease activity, and immunosuppressive therapy will be examined through subgroup, meta-regression, and sensitivity analyses when data permit. Certainty of evidence will be assessed using GRADE for diagnostic tests.

**Discussion:**

This review aims to determine whether commonly used and emerging host-response biomarkers retain adequate diagnostic performance in inflammatory arthritis, identify candidate population-specific thresholds that require prospective external validation, and clarify where evidence is insufficient for clinical implementation.

**Trial registration:**

PROSPERO CRD420261449928.

## 1 Introduction

Total hip and knee arthroplasty substantially improve pain and function in patients with advanced joint disease, but periprosthetic joint infection (PJI) remains one of the most consequential complications. This complication is associated with repeated surgery, prolonged antimicrobial treatment, impaired quality of life, high healthcare expenditure, and excess mortality. Because no single test can reliably establish or exclude infection, contemporary definitions combine clinical findings, microbiology, histopathology, serum inflammation markers, and synovial tests (1–3). The 2018 International Consensus Meeting definition and the 2021 European Bone and Joint Infection Society definition have improved diagnostic standardization; however, both require careful interpretation in populations with altered baseline inflammatory responses (1, 2).

Patients with inflammatory arthritis constitute one such high-risk population. Rheumatoid arthritis (RA), psoriatic arthritis, spondyloarthritis, systemic lupus erythematosus (SLE) with inflammatory joint involvement, and related immune-mediated diseases are characterized by dysregulated innate and adaptive immune activation. Disease activity can elevate C-reactive protein (CRP), erythrocyte sedimentation rate (ESR), circulating leukocyte-derived indices, and synovial leukocyte counts in the absence of infection. Conversely, glucocorticoids, conventional synthetic disease-modifying antirheumatic drugs, biologic agents, and targeted synthetic agents may attenuate fever, local inflammatory signs, cytokine production, or leukocyte recruitment. Immune dysregulation and pharmacologic immunosuppression also increase susceptibility to infection and may contribute to culture-negative or clinically indolent presentations (4–6).

The overlap between sterile autoimmune inflammation and infection creates two related diagnostic problems. First, thresholds developed mainly in osteoarthritis (OA) populations may not be directly translatable to patients with inflammatory arthritis. A universal threshold that is too low may misclassify an inflammatory flare or aseptic implant failure as PJI and lead to unnecessary revision surgery or antimicrobial exposure. A threshold that is too high may miss infection in an immunosuppressed patient. Second, the relative value of conventional markers and newer host-response biomarkers is uncertain. Serum CRP and ESR are inexpensive and widely available, whereas synovial white blood cell count, polymorphonuclear neutrophil percentage, leukocyte esterase, alpha-defensin, synovial CRP, calprotectin, interleukins, and other soluble immune mediators may provide different balances of sensitivity, specificity, cost, and accessibility.

Early prospective evidence suggested that conventional serum and synovial tests had broadly similar diagnostic utility in inflammatory and non-inflammatory arthritis, although reported thresholds differed (7). Subsequent studies have reported only moderate sensitivity for serum CRP and ESR when assessing persistent infection during staged revision (8), high accuracy for laboratory alpha-defensin in selected patients with systemic inflammatory disease (9), and substantially different study-derived cutoffs for CRP, ESR, synovial white blood cell count, and polymorphonuclear neutrophil percentage in RA compared with OA (10). A large retrospective cohort published in 2024 similarly reported higher study-derived CRP, ESR, and D-dimer thresholds but lower overall diagnostic performance in RA (11). These findings suggest clinically important threshold modification, but they arise from heterogeneous designs, reference standards, disease populations, infection stages, and index-test methods.

A 2019 systematic literature review identified a very limited evidence base and did not provide a contemporary diagnostic meta-analysis or formal certainty assessment (12). Since then, additional RA-specific cohorts, alpha-defensin data, and studies describing culture-negative and histologic phenotypes in inflammatory arthritis have been published (6, 9–11, 13). A 2025 narrative review highlighted persistent uncertainty and the need for adjusted diagnostic approaches but did not use systematic diagnostic meta-analytic methods (14). Meanwhile, broad PJI biomarker reviews generally combine inflammatory and non-inflammatory populations, potentially obscuring clinically relevant spectrum effects and incorporation bias (15, 16).

The proposed review will therefore evaluate the diagnostic performance and reported diagnostic thresholds of serum and synovial host-response biomarkers specifically in patients with inflammatory arthritis undergoing evaluation for hip or knee PJI. The review will prioritize paired sensitivity and specificity, address threshold and reference-standard heterogeneity, distinguish initial PJI diagnosis from assessment of persistent infection before reimplantation, and evaluate whether biomarkers perform differently in inflammatory arthritis and non-inflammatory comparator populations.

## 2 Objectives

The primary objective is to determine the diagnostic accuracy of serum and synovial host-response biomarkers for hip or knee PJI in adults with inflammatory arthritis.

Secondary objectives are to:

- estimate biomarker-specific sensitivity, specificity, positive and negative likelihood ratios, diagnostic odds ratios, and summary receiver operating characteristic (SROC) performance;
- identify reported diagnostic thresholds and assess whether thresholds differ from those used in general PJI definitions or in non-inflammatory comparator populations;
- compare serum and synovial biomarkers and, where direct within-study comparisons are available, compare conventional and emerging markers without relying on indirect cross-study rankings;
- examine whether diagnostic performance varies by inflammatory arthritis subtype, disease activity, immunosuppressive treatment, joint type, infection chronicity, diagnostic setting, and reference standard;
- assess the influence of incorporation bias, partial verification, case-control sampling, prior antimicrobial exposure, and other design features on diagnostic estimates; and
- rate the certainty of evidence and identify priorities for prospective immunologically informed diagnostic research.

## 3 Methods and analysis

### 3.1 Protocol development, registration, and reporting

This protocol was developed in accordance with the Preferred Reporting Items for Systematic Review and Meta-Analysis Protocols (PRISMA-P) statement (17). The completed review will be reported according to PRISMA 2020 and the PRISMA extension for diagnostic test accuracy reviews (PRISMA-DTA) (18, 19). Methods will also follow the Cochrane Handbook for Systematic Reviews of Diagnostic Test Accuracy (20). The protocol will be prospectively registered in PROSPERO before formal study selection. Any substantive amendment will be dated, justified, recorded in PROSPERO, and described in the final report.

The review will not begin outcome-driven data extraction or statistical synthesis before registration. Preliminary scoping searches used to assess feasibility and refine terminology will not be used as a substitute for the formal reproducible search.

### 3.2 Eligibility criteria

Eligibility criteria are summarized in Table 1 and described below.

**Table 1:** Eligibility criteria for the systematic review and diagnostic test accuracy meta-analysis.

Domain	Inclusion criteria	Exclusion criteria
Population	Adults with physician-diagnosed inflammatory arthritis or systemic inflammatory disease with inflammatory joint involvement, evaluated for suspected hip or knee PJI.	No extractable inflammatory-disease subgroup; no clinical confirmation of inflammatory condition where this cannot be clarified; pediatric-only cohorts.
Index tests	Serum, plasma, whole-blood, or synovial host-response biomarkers with sufficient data for diagnostic accuracy estimation.	Pathogen-detection tests alone; imaging alone; histopathology alone; undefined multimarker algorithms without separable host-response data.
Target condition	Initial hip or knee PJI or persistent infection before reimplantation, analyzed separately.	Noninfectious outcomes; native-joint septic arthritis; shoulder, elbow, ankle, or small-joint PJI only.
Reference standard	MSIS, ICM, EBJIS, IDSA, or another explicit composite clinical reference standard.	No explicit diagnostic criteria or unstructured clinician impression without adjudication details.
Study design	Prospective or retrospective cohort, cross-sectional, registry, or diagnostic case-control studies with a comparator and extractable 2 × 2 data.	Case reports; uncontrolled case series; reviews; editorials; animal or *in vitro* studies.
Report characteristics	Any date, country, language, or publication status; duplicate cohort reports linked.	Insufficient report after author contact; abstract-only report with no usable data.

#### 3.2.1 Population and clinical setting

We will include studies of adults aged 18 years or older with physician-diagnosed inflammatory arthritis or an immune-mediated systemic inflammatory disease with clinically documented inflammatory joint involvement who are evaluated for suspected PJI after total hip or total knee arthroplasty. Eligible conditions include RA, psoriatic arthritis, axial or peripheral spondyloarthritis, ankylosing spondylitis, adult survivors of juvenile idiopathic arthritis, SLE with inflammatory arthritis, and other clearly defined inflammatory arthritides. Studies including mixed populations will be eligible only when data for the inflammatory arthritis subgroup can be extracted separately or obtained from investigators. Studies that classify inflammatory disease solely from an unvalidated administrative code without clinical confirmation will be retained but evaluated for applicability concerns.

Both initial diagnostic evaluation of a painful or failing prosthesis and evaluation for persistent infection before reimplantation will be eligible, but these settings will not be pooled indiscriminately. Acute postoperative, acute hematogenous, and chronic PJI will be extracted separately whenever reported. Studies confined to shoulder, elbow, ankle, or small-joint arthroplasty will be excluded because inflammatory cell thresholds, microbiology, and test performance differ by implant location and the current evidence base is dominated by hip and knee arthroplasty.

#### 3.2.2 Index tests

Eligible index tests are host-response biomarkers measured in serum, plasma, whole blood, or synovial fluid. Conventional biomarkers include CRP, ESR, peripheral leukocyte count, neutrophil-to-lymphocyte ratio, platelet-related indices, fibrinogen, D-dimer, and procalcitonin; synovial tests include white blood cell count, polymorphonuclear neutrophil percentage, leukocyte esterase, alpha-defensin, synovial CRP, calprotectin, and cytokines or soluble immune mediators. Newly described protein, cellular, metabolomic, or composite host-response signatures will be eligible when evaluated against an explicit PJI reference standard and when sufficient data can be obtained to construct a 2 × 2 table.

Microbiological culture, sonication culture, broad-range polymerase chain reaction, metagenomic sequencing, and pathogen-specific assays will not be treated as index host-response biomarkers. Studies evaluating combined diagnostic algorithms will be included only when the contribution or performance of the host-response component is separable, or when the algorithm itself is explicitly defined as a clinically applicable host-response panel. Histopathology will be described as a reference-standard or complementary diagnostic component but will not be pooled with soluble or cellular biomarker tests.

#### 3.2.3 Reference standards

Acceptable reference standards include the Musculoskeletal Infection Society criteria, International Consensus Meeting criteria, European Bone and Joint Infection Society definition, Infectious Diseases Society of America criteria, or another prospectively specified composite reference standard incorporating clinical, microbiological, and operative findings (1–3). Studies using clinician diagnosis without explicit criteria will be included only if adjudication procedures are described and will be judged to have high or unclear risk of reference-standard bias. Because several candidate biomarkers are embedded within consensus definitions, we will record whether the evaluated index test formed part of the reference standard. Analyses excluding studies with incorporation bias will be prespecified.

#### 3.2.4 Target condition

The target condition is hip or knee PJI. Initial PJI and persistent infection at reimplantation are clinically distinct target conditions and will be synthesized separately. When a study reports both, separate 2 × 2 tables will be extracted. If this is impossible, the study will contribute to narrative synthesis only unless one setting accounts for at least 90% of the sample.

#### 3.2.5 Study designs

Prospective or retrospective cross-sectional and cohort diagnostic accuracy studies will be eligible. Diagnostic case-control or two-gate studies will also be eligible because the evidence base is expected to be limited, but they will be identified as high risk for patient-selection bias and excluded in sensitivity analyses. Comparative accuracy studies, nested cohorts, and secondary analyses of prospectively assembled registries will be included. Case reports, case series without a non-PJI comparison group, reviews, editorials, conference abstracts without sufficient data after author contact, animal studies, and *in vitro* studies will be excluded.

#### 3.2.6 Report characteristics

No restrictions will be applied by publication date, country, language, or publication status. Full reports in languages not understood by the review team will be translated by a qualified bilingual researcher or professional service. Preprints will be considered only if a complete methods section and extractable diagnostic data are available; if a peer-reviewed version becomes available before final analysis, the peer-reviewed report will be used. Multiple publications from the same cohort will be linked and treated as one study, with the most complete data extracted across reports.

### 3.3 Information sources

We will search MEDLINE via PubMed, Embase via Ovid, Web of Science Core Collection, Scopus, CINAHL, the Cochrane Library, China National Knowledge Infrastructure, Wanfang Data, VIP Database, and SinoMed from database inception to the date of the final search. ClinicalTrials.gov and the World Health Organization International Clinical Trials Registry Platform will be searched for ongoing or unpublished diagnostic studies. We will search PROSPERO to identify potentially overlapping reviews. Reference lists of included studies and relevant reviews will be examined, and forward citation tracking will be performed in Web of Science and Google Scholar. Corresponding authors will be contacted when subgroup data, thresholds, or 2 × 2 cell counts are missing.

The search will be updated within 30 days before the final analysis. Searches and deduplication will be documented sufficiently to reproduce the number of records retrieved from each source. The complete draft search strategies are provided in Supplementary File 1 and will be peer reviewed by a second information specialist using the PRESS guideline before execution (21).

### 3.4 Search strategy

The primary search will combine controlled vocabulary and free-text terms for joint arthroplasty or prosthetic joint infection with terms for inflammatory arthritis and immune-mediated rheumatic disease. Biomarker terms will not be mandatory in the primary search because eligible reports may emphasize the clinical diagnosis rather than name the index test in the title or abstract. A supplementary focused search will add terms for CRP, ESR, synovial leukocytes, alpha-defensin, calprotectin, cytokines, and other host-response biomarkers. A diagnostic accuracy filter will not be used because such filters may reduce sensitivity in a small and heterogeneous evidence base. Search syntax will be adapted to each database without language restrictions.

### 3.5 Record management and study selection

Search results will be imported into EndNote for deduplication and then into Rayyan or an equivalent systematic review platform. Two reviewers will independently screen titles and abstracts against predefined criteria. Before formal screening, the reviewers will pilot the criteria on at least 50 records and resolve interpretive differences. Records considered potentially eligible by either reviewer will undergo full-text assessment. Reasons for full-text exclusion will be recorded using mutually exclusive categories.

Disagreements will be resolved by discussion; unresolved disagreements will be adjudicated by a third reviewer. Inter-reviewer agreement will be reported for both screening stages using percent agreement and Cohen's kappa, but eligibility decisions will not be determined by a numerical agreement threshold. The final study selection process will be shown in a PRISMA flow diagram.

### 3.6 Data extraction

Two reviewers will independently extract data using a standardized, piloted form. The form will be tested on at least three eligible studies and revised before full extraction. Discrepancies will be resolved by consensus or third-reviewer adjudication. Extracted items are summarized in Table 2.

**Table 2:** Prespecified data items and planned use in synthesis.

Data domain	Items to be extracted
Study and setting	Author, year, country, center type, recruitment period, design, sampling method, prospective registration, funding, and conflicts of interest.
Population	Sample size, age, sex and/or gender as reported by the primary study, inflammatory arthritis diagnosis, disease-specific activity measure and definition, score or category, comorbidities, joint type, reason for revision, and PJI prevalence.
Immunomodulatory treatment	Glucocorticoid dose, conventional synthetic DMARDs, biologics by drug class or molecular target when reported, JAK inhibitors, timing of interruption or resumption, and other immunosuppression.
Target condition	Initial versus persistent infection, acute versus chronic PJI, culture-positive versus culture-negative infection, and microbiological spectrum.
Index test	Specimen, assay platform, timing, blinding, prespecified or data-derived threshold, units, handling of indeterminate results, and test failures.
Reference standard	Definition used, component criteria, adjudication, blinding, whether index test was incorporated, and timing relative to index sampling.
Accuracy data	True positive, false positive, true negative, false negative, sensitivity, specificity, likelihood ratios, diagnostic odds ratio, and AUC.
Comparative data	Within-study comparisons, non-inflammatory comparator results, threshold differences, and relative sensitivity or specificity.
Bias modifiers	Consecutive enrollment, case-control sampling, prior antibiotics, recent surgery, crystal arthropathy, metallosis, verification, missing data, and exclusions.

For each index test and reported threshold, we will extract true-positive, false-positive, true-negative, and false-negative counts. When cell counts are not reported, they will be reconstructed from sample size, prevalence, sensitivity, specificity, predictive values, or receiver operating characteristic (ROC) data when mathematically valid. Reconstruction assumptions will be documented and verified by a second reviewer. If several thresholds are reported, the prespecified clinical threshold will be prioritized; if no threshold was prespecified, all usable thresholds will be extracted and the data-dependent nature of any selected data-derived threshold will be recorded. Study authors will be contacted up to three times over six weeks for missing subgroup or threshold-specific information.

Inflammatory disease characteristics will include diagnosis, disease duration, the disease-specific activity measure and definition used in each study, rheumatoid factor or anti-citrullinated protein antibody status when available, glucocorticoid dose, conventional synthetic disease-modifying antirheumatic drugs, biologic agents by drug class or mechanism when reported, Janus kinase inhibitors, timing of medication interruption, and other immunosuppressive therapies. Original disease-activity scores or categories will be retained where possible, and cross-disease harmonization will be undertaken only when definitions are sufficiently comparable; otherwise, the influence of disease activity will be synthesized descriptively. These variables are central to the review because they may modify both infection risk and biomarker expression.

### 3.7 Outcomes

The primary outcomes are paired sensitivity and specificity for each host-response biomarker in inflammatory arthritis at a defined threshold. Co-primary presentation will include the summary point and 95% confidence region, together with the 95% prediction region when estimable.

Secondary outcomes are positive and negative likelihood ratios, diagnostic odds ratio, area under the ROC curve when appropriately estimated, reported diagnostic thresholds, and the difference in thresholds or diagnostic performance between inflammatory arthritis and non-inflammatory comparator groups. Where studies provide patient-level consequences, we will extract false-positive and false-negative classifications, changes in diagnostic category, and downstream management decisions. Predictive values will be reported descriptively because they depend on prevalence and clinical setting.

### 3.8 Risk of bias and applicability

Two trained reviewers will independently assess risk of bias and applicability using QUADAS-3, the current version of the Quality Assessment of Diagnostic Accuracy Studies tool (22). Before assessment, the reviewers will define the synthesis question and the ideal target test-accuracy study for each clinically distinct biomarker and diagnostic setting. Review-specific concerns will include non-consecutive or two-gate sampling, exclusion of diagnostically difficult or culture-negative cases, index thresholds selected after inspection of study data, lack of blinding, incorporation of the index biomarker into the reference standard, differential or partial verification, inappropriate flow and timing, and selective availability of threshold-specific estimates.

Studies making direct comparative claims between two or more biomarkers will additionally be assessed using QUADAS-C adapted for use alongside QUADAS-3, in accordance with developer guidance (23). The QUADAS-C tool will not be used as a stand-alone assessment. Reviewers will calibrate judgments on at least three studies. Domain-level and overall judgments will be reported without calculating a numerical quality score. Applicability will be judged against the target population of adults with inflammatory arthritis undergoing real-world evaluation for hip or knee PJI.

### 3.9 Data synthesis and statistical analysis

#### 3.9.1 Descriptive synthesis

All eligible studies will be summarized in tables grouped by specimen type, biomarker, diagnostic setting, and reference standard. Study flow, inflammatory disease spectrum, immunosuppressive treatment, assay methods, thresholds, and 2 × 2 data will be displayed. Forest plots of sensitivity and specificity will be generated for each biomarker even when statistical pooling is not appropriate. Threshold distributions will be summarized using medians, ranges, and clinically relevant reference values.

#### 3.9.2 Quantitative synthesis

Meta-analysis will be considered when at least four studies evaluate the same biomarker in a clinically coherent diagnostic setting and provide usable paired accuracy data. Sensitivity and specificity will not be pooled separately using conventional univariate methods. When studies use a common or sufficiently similar threshold, a bivariate random-effects model will jointly synthesize logit sensitivity and specificity (24). When thresholds vary materially, a hierarchical summary receiver operating characteristic model will be used to characterize the accuracy-threshold relationship (25). Models will be fitted using Stata and R with established diagnostic meta-analysis packages. Exact binomial likelihood methods will be preferred; a continuity correction will not be applied routinely to zero cells.

Initial diagnosis and persistent infection before reimplantation will be analyzed separately. Serum and synovial measurements of the same analyte will also be treated as distinct index tests. Different assay formats, such as laboratory alpha-defensin immunoassay and lateral-flow alpha-defensin, will not be combined unless clinical and analytical equivalence is justified. If fewer than four studies are available or models are unstable, findings will be synthesized narratively without presenting a potentially misleading pooled estimate.

#### 3.9.3 Comparative accuracy

Direct within-study comparisons will be prioritized because indirect comparisons across separate study sets are vulnerable to spectrum and threshold confounding. Where sufficient direct comparative data exist, comparative accuracy will be assessed using hierarchical meta-regression with test type as a covariate or by estimating relative sensitivity and specificity. We will not rank biomarkers solely according to separate pooled diagnostic odds ratios or areas under unrelated summary curves.

#### 3.9.4 Heterogeneity and threshold effects

Clinical and methodological heterogeneity will be evaluated from forest plots, SROC plots, prediction regions, between-study variance estimates, and prespecified covariates. The I-squared statistic will not be used as the principal measure of heterogeneity for paired diagnostic accuracy because variability in sensitivity and specificity is strongly influenced by threshold effects and the statistic has limited interpretability in this setting.

If at least ten studies are available for a biomarker, meta-regression may examine inflammatory arthritis subtype, disease activity using disease-specific measures or categories only when sufficiently comparable across studies, systemic glucocorticoid exposure, individual immunomodulatory drug classes or mechanisms when adequately reported, hip versus knee arthroplasty, acute versus chronic PJI, initial diagnosis versus reimplantation, prospective versus retrospective design, consecutive versus case-control sampling, reference standard, assay format, and presence of incorporation bias. When disease-activity definitions or medication exposures cannot be harmonized without substantial misclassification, their influence will be summarized descriptively rather than forced into meta-regression. The number of covariates will be limited to avoid overfitting, and subgroup results will be interpreted as exploratory unless based on direct prespecified comparisons.

#### 3.9.5 Sensitivity analyses

Where data permit, analyses will be repeated after:

- excluding diagnostic case-control or two-gate studies;
- excluding studies judged at high overall risk of bias using QUADAS-3 or at high risk in domains most relevant to the synthesis;
- excluding studies in which the index biomarker contributed to the PJI reference standard;
- restricting to RA;
- restricting to chronic hip or knee PJI evaluated before revision surgery;
- restricting to studies with a recognized consensus reference standard;
- excluding reconstructed 2 × 2 tables or author-selected data-derived thresholds; and
- excluding studies in which recent surgery, crystal arthropathy, metallosis, or antimicrobial exposure could not be separated.

#### 3.9.6 Small-study effects and reporting bias

When at least ten studies contribute to a biomarker analysis, small-study effects will be explored using Deeks’ funnel plot asymmetry test (26). Results will be interpreted cautiously because selective threshold reporting, case-control designs, and heterogeneity may produce asymmetry in the absence of publication bias. Trial registries, conference records, and protocols will be compared with publications to identify non-publication or selective reporting where possible.

### 3.10 Certainty of evidence

The certainty of evidence for key biomarker-threshold combinations will be assessed using GRADE guidance for diagnostic tests and strategies (27). Evidence will be evaluated for risk of bias, indirectness, inconsistency, imprecision, and publication bias. Judgments will consider whether accuracy estimates are linked to clinically important consequences, including unnecessary revision or antimicrobial treatment after false-positive results and delayed treatment after false-negative results. Certainty will be rated as high, moderate, low, or very low, with explicit reasons for each judgment. Summary of Findings tables will present anticipated true-positive, false-negative, true-negative, and false-positive results at clinically relevant PJI prevalences.

### 3.11 Patient and public involvement

Patients and members of the public were not involved in development of this protocol. The completed review will emphasize patient-important consequences of diagnostic error and will be disseminated in an accessible summary suitable for patients considering revision arthroplasty.

### 3.12 Review status and anticipated timeline

At submission of this protocol, formal screening, outcome-driven data extraction, and meta-analysis will not have been completed. After protocol registration, the planned sequence is database searching and deduplication, title and abstract screening, full-text assessment, data extraction and risk-of-bias assessment, statistical synthesis, and preparation of the final report. Dates will be updated in PROSPERO if the project timeline changes.

## 4 Discussion

This protocol addresses a diagnostic problem at the interface of autoimmunity, infection, and arthroplasty. In patients with inflammatory arthritis, the same host-response pathways used to detect PJI may already be activated by autoimmune disease or suppressed by treatment. A biomarker may therefore have excellent performance in a general arthroplasty population yet yield clinically unacceptable misclassification in a patient with active RA or recent biologic therapy. By restricting the primary question to inflammatory arthritis and extracting immunologic and treatment-related modifiers, the review is designed to evaluate generalizability to inflammatory arthritis rather than simply reproduce broad PJI biomarker estimates.

The review has several methodological strengths. It will use paired diagnostic meta-analysis rather than separate pooling of sensitivity and specificity; distinguish initial PJI from persistent infection at reimplantation; treat serum and synovial measurements and distinct assay formats separately; and explicitly assess incorporation bias when biomarkers are embedded within the reference standard. Direct comparisons will be prioritized over indirect rankings. In addition, the review will collect disease-specific activity measures and definitions and immunomodulatory treatment by class or mechanism when reported, variables frequently omitted from conventional orthopedic evidence syntheses despite their biological relevance.

The likely limitations arise from the underlying evidence. Inflammatory arthritis cohorts are uncommon, single-center retrospective designs predominate, and studies may group heterogeneous immune-mediated diseases. Reference standards have evolved over time, and older studies may use definitions that differ materially from current practice. Data-dependent threshold selection can exaggerate apparent accuracy, while inclusion of the index test in a composite reference standard can create incorporation bias. Disease activity, medication exposure, recent antibiotics, and timing of specimen collection may be poorly reported. These limitations may preclude pooling for some biomarkers and will probably reduce certainty of evidence. Because the available evidence may be dominated by RA, thresholds derived largely from RA will not be assumed to apply to other inflammatory arthritides. Disease-activity measures differ across conditions, and medication effects may vary by drug class or mechanism; sparse or inconsistently reported data may prevent valid harmonization or drug-class-specific analyses.

The review will not assume that a statistically selected threshold is automatically clinically preferable. Threshold selection must reflect the relative consequences of missed infection and unnecessary treatment, which vary across preoperative screening, revision planning, and reimplantation settings. Where evidence permits, findings will therefore be presented at clinically relevant prevalence values and interpreted according to the expected numbers of false-positive and false-negative results. This approach may help identify biomarkers suitable for ruling out infection, ruling in infection, or prompting additional testing rather than serving as stand-alone tests. Any candidate threshold identified by the review should therefore be regarded as hypothesis-generating until it has undergone prospective external validation in an independent clinical cohort.

The findings may have implications for future consensus definitions and study design. If conventional thresholds perform differently in inflammatory arthritis, validation cohorts should prespecify disease-specific thresholds and report disease activity and immunomodulatory treatment. Emerging biomarkers should be evaluated in consecutive, prospectively enrolled patients and compared directly with established tests using a reference standard that minimizes incorporation bias. Multicenter collaborations will be required to obtain adequate numbers of confirmed PJI cases across different inflammatory diseases and treatment regimens.

## 5 Ethics and dissemination

Ethics approval and informed consent are not required because this review will use aggregate data from published or publicly accessible studies and will not collect identifiable patient information. The review will be conducted in accordance with established standards for research integrity and transparent reporting. Results will be submitted to a peer-reviewed journal, presented at orthopedic, rheumatology, infectious disease, or immunology meetings, and summarized for clinical audiences. The final search strategies, extraction form, analytic code, and study-level dataset will be deposited in an open repository when permitted by source-report copyright and data-use conditions.

## Data Availability

The original contributions presented in the study are included in the article/Supplementary Material, further inquiries can be directed to the corresponding authors.
